# The Importance of Research Experience With a Scoreless Step 1: A Student Survey at a Community-Based Medical School

**DOI:** 10.7759/cureus.43476

**Published:** 2023-08-14

**Authors:** Nicholas P Radulovich, Skyler Burke, Nathan J Brown, Brett Jones, James Antongiovanni, Douglas Nanu, John Roll

**Affiliations:** 1 Ophthalmology, Washington State University Elson S. Floyd College of Medicine, Spokane, USA; 2 Oncology, Washington State University Elson S. Floyd College of Medicine, Spokane, USA; 3 Medicine, Washington State University Elson S. Floyd College of Medicine, Spokane, USA; 4 Research, Washington State University Elson S. Floyd College of Medicine, Spokane, USA

**Keywords:** community-based medical school, residency application, medical research, medical student perspective, usmle step 1

## Abstract

Purpose: As of January 26, 2022, the United States Medical Licensing Examination (USLME) step 1 exam went from a scored test to pass-fail step 1 (PFS1). The authors were interested in surveying medical students at a community-based medical school to observe their perceptions of the importance of student research given this recent change.

Method: A Qualtrics survey was disseminated to medical students (years 1-4) via school emails. Data were analyzed using the Mann-Whitney test to assess Likert scale scores, and narrative comments were grouped as qualitative feedback. Survey dissemination and analysis of data were both conducted at a large community-based medical school.

Results: The survey sampled 104 students categorized into pre-clerkship (PC) and clerkship (CL) years, with a response rate of 33%. A contradiction was found, as indicated by the higher number (*p *= 0.047) of clerkship students interested in Primary Care/Family medicine residency compared to pre-clerkship students at 41% and 59%, respectively. Whereas participants who indicated they are interested in pursuing a competitive specialty for residency were 51% of pre-clerkship students over 41% of clerkship students (*p *= 0.047)*. *Additionally, given the assessment change to pass/fail, students did in fact believe that residencies would now view research as a higher assessed component than before (79% pre-clerkship and 72% clerkship). However, a minority of students said that they increased their research efforts (41% and 47%). Most students supported the research opportunity improvements proposed in our survey.

Conclusions: Efforts to make the step 1 exam pass/fail may have alleviated some stress related to performance but may have increased the perception of the importance of other components in a student’s residency application. Our survey highlights how medical students at a community-based medical school perceive this change and how it has affected their research efforts.

## Introduction

As of January 26, 2022, the United States Medical Licensing Examination (USLME) step 1 (S1) exam transitioned from a scored test to a pass-fail step 1 (PFS1). The purpose was to promote a more holistic residency evaluation process and address concerns about student well-being. Since this examination has long been considered a critical metric in determining which applicants to both interview and rank, uncertainty exists among medical students and residency program directors moving forward with regards to preparing a competitive application [1]. While many have questioned whether a scored step 2 exam will take the place of a PFS1 when deciding residency fit, that does not align with the efforts by the USMLE to create a more holistic residency evaluation process [2].

Another metric that is making its way into national discussions is research involvement [3]. Engagement in research can provide opportunities to learn more about a particular field, demonstrate dedication, network, and foster an early interest in contributing to the primary literature. In the context of shifting to a holistic review process, research engagement is thought by some to be increasingly important. However, few studies exist examining these perceptions, especially among medical students. This study seeks to better understand the attitudes toward and engagement with research among medical students at a community-based medical school.

Given this recent change, we sought to also compare the perceptions of first- and second-year medical students, or pre-clerkship (PC) students, and third- and fourth-year or clerkship (CL) students on their perceptions. We know that residency programs, especially the more competitive ones, prioritize research experiences as one of their criteria for acceptance [4]. Therefore, looking at a student's specialty of interest and their perception of research can help us determine if there is a disconnect between what is preferred in terms of the number of research experiences or a lack of resources to gain these research experiences for community-based medical students. Student surveys will address components such as the importance of doing research while in medical school, a specialty of interest, and a detailed assessment of engagement in research to date.

This research is sponsored by the Graduate Education Academic Research Society (GEARS) at the medical school. GEARS is a group focused on improving access to impactful education, research, and collaboration opportunities for all students. Its mission is to promote the engagement of interdisciplinary research through advocacy, project initiatives, interprofessional research education, and research grand rounds.

## Materials and methods

Prior literature on the topic was reviewed before conducting this study. A comprehensive literature review was performed, and a total of 11 reports related to the objectives of this study and/or study design were found using PubMed and the search terms: USMLE step 1, medical student perspective, medical research, residency application. Ethical approval for this study was obtained by the institution's Human Research Protection Program (HRPP), IRB # 19767-001.

Study design

An electronic survey was developed using the Qualtrics XM Platform (Qualtrics, Provo, UT), and a survey link was sent to first- to fourth-year medical students via their academic email. Survey respondents were broken down into two groups: pre-clerkship and clerkship. Students in the pre-clerkship group have yet to take the USMLE Step 1 examination, and those in the clerkship group have already taken the examination. Students who did not fall into either of these groups were asked not to participate in the recruitment email. A three-week period was allotted for students to electively complete the 16-question survey. Respondent answers were kept anonymous, survey completion was optional, and students had the capability to stop the survey at their leisure. All participants agreed electronically to informed consent prior to filling out the survey.

The survey consisted of both multiple-choice and short-response answers. Most of the survey questions addressing the study objectives were in Likert-scale format. Students and faculty members involved in graduate medical programs assessed and modified survey questions for clarity.

The survey questions asked about participant demographic information such as gender, age, and year in medical school. Additional questions inquired about students’ perspectives on the importance of research in medical school, their medical specialty of interest, and their thoughts on the weight research may carry for their specialty of interest residency application. Participants' past research experience, desire to work in under-served rural areas as a future provider, and thoughts on how residency programs may emphasize research experience were also gaged. 

Response analysis

Quantitative methods were utilized in analyzing the results of this study. Descriptive statistics were used to describe demographics, the research background of students, and their intentions to practice medicine in rural, underserved communities.

The remaining survey responses were split into two student groups: pre-clerkship (first- and second-year medical students) and clerkship (third- and fourth-year medical students). This grouping better reflects the different objectives and learning stages of each class. P-values were calculated using a Mann-Whitney non-parametric test to compare the 5-point Likert scale responses of pre-clerkship and clerkship students. Statistical analysis was completed using SPSS Software version 28.0 (SPSS Inc., Chicago, IL, USA). Mann-Whitney tests were used to assess survey responses between pre-clerkship and clerkship students, in which Likert scales were used.

Respondent comments were pulled to assess students’ attitudes and common themes as they pertain to the study objectives.

## Results

Three hundred and twenty students were emailed a survey link, of which 104 completed the survey, with a response rate of 33%. The majority of the 104 respondents were MS2s at 37/104 (35%), followed by MS1s at 28/104 (27%). Then MS3 and MS4 participation in the survey was 22/104 (21%) and 17/104 (16%), respectively. The majority of respondents were 25-29 years old, and there was a 3:2 male-to-female respondent ratio. These results and additional demographic data can be found in Table 1. Being that this is a community-based medical school with an emphasis on selecting students from rural and underserved areas, it was expected that a considerable portion of the class intends to practice medicine in a rural or underserved community [5].

**Table 1:** Overall response rate basic demographics M1-4: medical year 1-4

Demographic (n)	Survey response, n (%)
Age (104)	
Under 25	17 (16%)
25–29	63 (61%)
30–34	18 (17%)
35+	6 (6%)
Gender (104)	
Male	61 (59%)
Female	41 (39%)
Prefer not to answer	2 (2%)
Year in school (104)	
M1	28 (27%)
M2	37 (36%)
M3	22 (21%)
M4	17 (16%)
Intention to practice medicine in the rural or underserved community	
M1 (28)	10 (37%)
M2 (37)	13 (35%)
M3 (22)	8 (37%)
M4 (17)	2 (14%)

Our survey data were grouped among pre-clerkship and clerkship students, as this allowed for direct comparison of differences or similarities in opinions between cohorts of medical students at each important phase of their education. These comparisons are highlighted in Table 2. Survey response retention was slightly diminished in both PC and CL groups. Based on this understanding of experience from years in medical schools, the average research-related deliverables such as poster presentations, podium presentations, and publications are significantly higher in every category when comparing PC and CL students in Table 2.

**Table 2:** Survey questions regarding the career aspirations of participants *Mann-Whitney test between responses of 5-point Likert scale

	Pre-clerkship students (65)	Clerkship students (39)	P-value for trend*
Interested in primary care/family medicine residency, n (%)	31 (41%)	23 (59%)	0.047
Interested in a ‘competitive’ specialty for residency, n (%)	33 (51%)	16 (41%)	0.047
Number of research-related deliverables (mean)			
Poster presentation	0.61	3.03	0.004
Podium presentation	0.54	1.9	0.005
Peer-reviewed publication	0.63	2.24	0.035
Intending to pursue a career in academic medicine and/or will continue to be involved in research throughout medical career, n (%)	21 (33%)	12 (30%)	0.191

Additionally, our analysis indicated a significantly higher number (p = 0.047) of CL students who are interested in Primary Care/Family medicine residency compared to PC students at 41% and 59%, respectively. This contrasts with participants who indicated they are interested in pursuing a competitive specialty for residency, in which 51% of PC students agreed over 41% of PL students (p = 0.047) (Table 2). Competitive specialties are defined in no particular order as plastic surgery, otolaryngology, neurological surgery, vascular surgery, orthopedic surgery, interventional radiology, and urology per the National Resident Matching Program (NRMP) Charting Outcomes in the Match Data [6]. There was no significant difference between the two cohorts in terms of intent to pursue a career in academic medicine or continue to produce research (p = 0.191) (Table 2).

There were no significant differences between the two cohorts for the questions on the importance of research in the specialty of interest, the increase in research with step 1 going to pass-fail, the importance of a dedicated research project (e.g., a capstone), the views of residency programs on research experiences with a pass-fail step 1, and the opinions of inter-professional research projects (Table 3). Despite there being no objective difference between cohorts, a majority of students in both groups ‘Agreed’ or ‘Strongly Agreed’ to the later questions, apart from an overall disagreement regarding the importance of increasing research experiences with a pass-fail score in step 1. Furthermore, 52/58 (90%) of PC students agreed that research was an important experience for their specialty of choice, while not significantly different but approaching significance (p = 0.077) only 24/33 (73%) of CL students agreed.

**Table 3:** Survey questions regarding student interpretations of research importance PC: pre-clerkship; CL: clerkship *Responses 4 and 5 in 5-point Likert scale were grouped as 'agreement' for reporting purposes **Mann-Whitney test between responses of 5-point Likert scale ***Fourth-year medical students excluded from question as they took a scored step 1 exam

Survey questions	Agreement* from students of PC status (58)	Agreement* from students of CL status (33)	P-value for trend**
Importance to have research related experience in specialty of interest, n (%)	52 (90%)	24 (73%)	0.077
Did you increase your research commitments in anticipation of step 1 switching to pass/fail?***, n (%)	24 (41%)	9/19 (47%)	0.4
Do you feel it is beneficial to have a required research project as a component of your medical school curriculum?, n (%)	40 (69%)	23 (69%)	0.797
Do you feel residency programs will view research experiences more important as step 1 is moved to Pass/Fail?, n (%)	46 (79%)	24 (72%)	0.436
Do you feel it is important to have inter-professional collaborative research? (i.e., working with students from other colleges (nursing, pharmacy, etc.), n (%)	41 (70%)	21 (63%)	0.819

Specific questions focused on how the results of the survey could be implemented within a community-based medical school (Table 4). The results noted significant differences among the PC and CL cohorts when discussing specific research-related programs or interventions. Significance was seen when asking if PC and CL students had adequate opportunities to pursue research projects, with the PC students agreeing at a rate of 12% (7/58) while CL agreed at 36% (12/33) (p = 0.023). Most PC students indicated they would participate in a journal club at 62% (36/58) while a minority of CL students affirmed at 39% (13/33) (p = 0.014), and similarly, almost all PC students indicated an interest in the research matching program at 96% (56/58) and a significant majority of CL students agreed at 75% (25/33) (p = 0.002). There was also majority interest in a related research project database, with 88% (51/58) of PC students and 72% (24/33) of CL students in favor, with no significant difference between the two groups (p = 0.053) (Table 4).

**Table 4:** Survey questions regarding expanding the availability of research and other miscellaneous resources PC: pre-clerkship; CL: clerkship

Survey questions	Agreement* from students of PC status (58)	Agreement* from students of CL status (33)	P-value for trend**
Did you have adequate opportunities to find a mentor in your specialty of interest?, n (%)	18 (31%)	14 (42%)	0.104
Are there adequate opportunities to purse research in your specialty of interest?, n (%)	7 (12%)	12 (36%)	0.023
How likely would you be to participate in a research journal club?, n (%)	36 (62%)	13 (39%)	0.014
How likely would you be to participate in a research matching program? (e.g., connecting students with available, interest-specific research projects), n (%)	56 (96%)	25 (75%)	0.002
How likely would you be to use a student-run research project database? (e.g., website with on-going research opportunities for students), n (%)	51 (88%)	24 (72%)	0.053

In terms of specific comments from students, as seen in Table 5, responses indicated that students felt that a blog or website of research opportunities would be beneficial to their career goals, aligning with responses detailed in Table 4. Additionally, one student remarked that they felt at a disadvantage when compared with larger, research-focused medical schools in terms of finding research projects and mentors. Multiple respondents mentioned they wanted specific specialty-aligned faculty to arrange specific research meetings to assist with this mentor-matching project.

**Table 5:** Narrative student comments (common theme selection) MS1: first-year medical student

Narrative comments
“I think it would be neat to have a website or even blog dedicated for [medical] students to submit small entries into what type of research they completed during their time [in medical school], how they got involved, and what they felt they got out of it. This would be a nice resource for incoming MS1's to learn more about what has been done in the past and what opportunities or research interests they might not have considered.”
“It would be helpful to have dedicated research faculty who can direct you to projects in your specialty.”
“More knowledge on the general progression of research (i.e., how to get funding, how to draft certain types of research manuscripts, how to get your abstract accepted for a conference, and how to get your paper published).”
“I think we should have a personalized research meeting with someone that can point us in the right direction and help us take actionable steps.”
“Having a more centralized [research] database that physicians in the community actually use and are aware of would be super helpful.”
“More specialty-specific research opportunities.”

## Discussion

The shift from a scored USMLE S1 to a PFS1 has been a considerable change in the landscape of medical education. Beyond simply changing how students prepare for the PC years, it has had a cascading impact on the trajectory of every year of medical education, from admission all the way to match. The original impetus of the change to PFS1 was to encourage students to focus more on developing teamwork, communication, and leadership traits while focusing less emphasis on a three-digit score [7]. In particular, the USMLE states they are prioritizing a new ideal system of holistic candidate evaluation and viewing the new PFS1 as a true benchmark achievement rather than a stratification tool for student evaluation [8]. The implications of this change are widespread and evolving. Some have praised the change for addressing known inequities in testing among underrepresented medicine (UiM) students [9], while others have indicated that the move is unpopular among osteopathic medical students in particular as it may give those students less of a chance to stand out to residency match committees and thus prefer a scored exam [10]. Regardless, this change is an indication of the shifting landscape of US medical education, further exemplified by the recent news that many top medical schools were withdrawing from the US News and World Report rankings of the best medical schools, stating that the evaluation was of inflated significance and led to more inequity and prejudice among those not included [11].

As attendees of a community-based medical school, this group sought to explore these dynamics in our survey project described here. Respondents were grouped under the presumption that CL students usually have already completed USMLE step 1 upon survey completion, while PC students have yet to take the step 1 exam. In general, PC medical students are typically focused on learning foundational medical knowledge and skills and may not be involved with research during this time. Nevertheless, PC students still pursue research projects to learn more about a specific area of medicine while building their resumes. In contrast, CL medical students are more advanced in their medical training and may be in the stage of conducting more research projects. Research experience can help medical students develop critical thinking and analytical skills, which are essential for any medical specialty. Additionally, research experience can help students understand how to evaluate evidence and make informed decisions about patient care. For these reasons, residency programs have historically appreciated some pursuance of research.

Initial comparison of PC and CL cohorts regarding career preferences (Table 2) showed that significantly more CL students are interested in Primary Care and Family Medicine residency compared to PC students, at 41% and 59%, respectively (p = 0.047). In terms of interest in more competitive (e.g., surgical) specialties, 51% of pre-clerkship students indicated interest compared to 41% of clerkship students (p = 0.047). One explanation of these findings could be that a PFS1 may mean that more students are interested in more competitive specialties early on now that they know an upper percentile score is no longer an unofficial prerequisite to applying. In contrast, CL students may think that they are less competitive without a high score on S1 prior to residency applications. These more competitive specialties have faced criticism for lacking diversity [12], with some theorizing that high S1 scores are an unintended barrier to increasing diversity and representation in these fields [13]. In theory, there should now be space for an influx of a more diverse application pool into these top fields with a PFS1. Alternatively, critics would argue that with a PFS1, there will be less opportunity for UiM students, students from osteopathic or community-based schools, or those from smaller, lesser-known medical schools to have a chance to get a residency interview invite without a stellar S1 score [10].

There was a significant difference in the average number of research deliverables between the PC and CL groups. This is aligned with the difference in the amount of exposure to research opportunities and completed projects that come in the third and fourth years, as well as more clarification in terms of career interest. In these results, we also see that there is no difference in terms of the importance of academic research in future careers among PC and CL students (Table 2). This suggests that around a third of both cohorts understand and emphasize the impact of academic medicine in both their early medical education and their future training and careers. These results are in contrast with the subsequent results (Table 3), which showed no difference between PC and CL students when considering the importance of increasing their research output in a PFS1 environment and the perceived importance of increased research achievements to residency programs. This suggests that both cohorts equally understand the importance of increasing research production in the setting of a PFS1. Of note, almost 80% of PC students felt that they viewed research experiences as more important with the new PFS1 and understood the implications of changing extracurricular commitments in a PFS1 environment. With the change to PFS1, program directors across the country have started to hint at the increased weight that research will have on students’ applications. Rachel et al. found that 41% of program directors will be more reliant on applicants' research participation. Importantly, they also found that 30% of respondents from community-based residency programs will take a closer look at research participation to evaluate students with the move to PFS1 [14].

As it relates to improving research output and therefore competitiveness in a PFS1 match world, these authors believe that one of the most important findings from this project is explored in the final quantitative results table (Table 4). Less than 50% of PC and CL students reported that they had adequate opportunities to find a mentor in their specialty of interest and that they had adequate opportunities to pursue research in their specialty of interest. Our findings are similar to those of a large study involving medical students and publication output in which Nguyen et al. found that students who attended the top 40 NIH-funded medical schools had higher numbers of publications when compared to those who did not attend the top 40 medical schools, including community-based medical schools [15]. Given this, several studies further the notion that students participating in dedicated research programs and with a higher number of publications [16,17] are more likely to receive invitations to interview [18] and to match [17,19-22] in certain specialties. Thus, community-based medical schools should consider implementing more intensive research mentorship and research initiatives. Some of the solutions we proposed to the survey recipients were a research journal club, a dedicated research mentor matching program, and a student-run research database. Among PC students, the dedicated research matching program and the student-run research project database were very favorably supported, with 96% and 88% of PC students indicating interest, respectively. A lesser but still favorable percentage of CL students also supported the idea, at 75% and 72%, respectively. One explanation for the significant difference in responses from PC and CL students to these questions can be explained by the difference in free time between PC and CL students as well as the significantly increased research production of CL students, indicating they may feel that research is less important to them now that they have several completed projects.

Prior to conducting this study, the authors of this project founded a new registered student organization titled GEARS, which was designed to lower the barrier of entry into research experiences for medical students at a new community-based medical school. With that goal in mind, we set out to simultaneously survey the student body with the results shared here and to also set out to implement a few data-driven solutions. The first is to establish an interdisciplinary journal club that would include the Colleges of Pharmacy, Nursing, and Nutrition Sciences. By setting this journal club up, our goal is to encourage interdisciplinary collaboration on research projects and to establish this medical school as a research organization with a community-focused mission. Additionally, we established a research mentor and project-matching database where students can browse for research opportunities and establish connections in their specialties of interest.

Limitations

The results of this study should be held in the context of our limitations: the small sample size poses a limitation on how externally valid our findings are. The data are additionally unique in that respondents are enrolled at a community-based medical school with a mission that emphasizes providing care to rural and underserved communities; this contrasts with US MD schools that are urban-based or research-focused. Additionally, our survey is susceptible to recall bias and neutral responses given how busy medical students are with minimal time to complete surveys. At the time of our survey, an unquantified percent of fourth-year students had matched into their chosen specialty, which may have also affected how perceptions were skewed. Opinions and perceptions are also not entirely independent amongst the two cohorts, as feedback and mentorship are often passed down to pre-clerkship students. Despite these limitations, our survey both quantitatively and qualitatively addresses important factors for students, medical schools, and residency programs to consider given that the USMLE is now pass/fail.

## Conclusions

Historically, step 1 scores were a major contributing factor in determining how competitive students were for residency programs. However, since step 1 is no longer quantified, this has changed. The purpose of this survey was to assess the perceptions and attitudes of medical students toward research once USMLE step 1 became a pass/fail exam. The results indicated that pursuing research is highly dependent on students' interests, specific residency program interests, and the time and resources available to them. Competitive specialties view research experience as a vital component of an applicant’s portfolio; however, students interested in non-competitive specialties may not need to invest as much time and effort into research. The results also suggested that medical students may benefit from more accessible, specialty-specific research opportunities to engage in research activities effectively. However, due to the study's small sample size and the recent change in the USMLE step 1 exam to pass or fail, further research needs to be done regarding medical student perceptions and the importance of research experiences. Similar survey-based studies conducted by other community-based medical schools and comparative studies by research-focused institutions may help define a conclusive narrative surrounding this topic.
